# Earlier social information has a stronger influence on judgments

**DOI:** 10.1038/s41598-023-50345-4

**Published:** 2024-01-02

**Authors:** Alan Novaes Tump, David Wollny-Huttarsch, Lucas Molleman, Ralf H. J. M. Kurvers

**Affiliations:** 1 Center for Adaptive Rationality, Max Planck Institute for Human Development, Berlin, Germany; 2 Exzellenzcluster Science of Intelligence, Technical University Berlin, Berlin, Germany; 3 Amsterdam Brain and Cognition, University of Amsterdam, Amsterdam, Netherlands; 4 Tilburg School of Social and Behavioral Sciences, Tilburg University, Tilburg, Netherlands

**Keywords:** Psychology, Human behaviour

## Abstract

People’s decisions are often informed by the choices of others. Evidence accumulation models provide a mechanistic account of how such social information enters the choice process. Previous research taking this approach has suggested two fundamentally different cognitive mechanisms by which people incorporate social information. On the one hand, individuals may update their evidence level instantaneously when observing social information. On the other hand, they may gradually integrate social information over time. These accounts make different predictions on how the timing of social information impacts its influence. The former predicts that timing has no impact on social information uptake. The latter predicts that social information which arrives earlier has a stronger impact because its impact increases over time. We tested both predictions in two studies in which participants first observed a perceptual stimulus. They then entered a deliberation phase in which social information arrived either early or late before reporting their judgment. In Experiment 1, early social information remained visible until the end and was thus displayed for longer than late social information. In Experiment 2, which was preregistered, early and late social information were displayed for an equal duration. In both studies, early social information had a larger impact on individuals’ judgments. Further, an evidence accumulation analysis found that social information integration was best explained by both an immediate update of evidence and continuous integration over time. Because in social systems, timing plays a key role (e.g., propagation of information in social networks), our findings inform theories explaining the temporal evolution of social impact and the emergent social dynamics.

## Introduction

Many, if not most, of people’s day-to-day decisions in domains as widespread as investment, voting, and health care are made in a social context, be it physical or virtual^1,2^. Taking the decisions of others into consideration can help to increase the accuracy of judgments^3–6^. Research on social information use has suggested two fundamentally different cognitive mechanisms by which decision makers can incorporate such social information. On the one hand, a decision maker can instantly update their level of subjective evidence in line with the social information (e.g., the decision of a majority;^7–9^). On the other hand, a decision maker can gradually align their level of subjective evidence toward the social information over time^10–12^. The two mechanisms imply different predictions about how the timing of social information will influence judgments.

Imagine you are considering buying a new smartphone online. You evaluate the quality of the various devices on offer by reading their descriptions. You also receive input from a friend who has recently invested in a new phone. Would this social information impact your judgment more if it were to arrive early or late in the decision process? According to the first account, the timing should not matter: You will instantaneously update your evidence in favor of the option indicated by the social information. According to the second account, in contrast, a rating received earlier in the evaluation process will have a stronger influence on your decision because it will continuously enter the evaluation process. Despite these opposing accounts, little research has yet investigated how the timing of social information impacts its uptake. However, as timing plays a key role in the propagation of information in social systems, understanding the role of timing on the uptake of social information is crucial for explaining the spread of beliefs and emerging collective behaviour in social systems such as on social media platforms. Here, we investigate whether the timing of social information influences its uptake in two experimental studies.

A powerful framework for investigating how individuals incorporate social information is the Judge–Advisor System (JAS;^2,13^). In this framework, the decision maker (i.e., the ‘judge’) evaluates task-specific information while receiving additional social information, typically the judgments of one or several other people (i.e., the ‘advisors’). Research on advice taking has identified several factors that impact the extent to which judges integrate such social information. For example, higher perceived expertise of the advisor and (monetary) incentives generally increase advice taking by judges and judges typically prefer opinions that are closely aligned with their own opinions^6,14,15^. The number of advisors, their confidence in their judgments and the presence or absence of interactions between advisors and judges are also known to impact the uptake of social information^13,16,17^. In most of these studies, the social information was provided at a fixed time point (but see^18^) and there was no consideration of the potential effects of when (timing) or for how long (duration) social information was presented. It therefore remains unclear how social information enters the choice process dynamically over time. Evidence accumulation models, which describe the temporal dynamics of the choice process, can help to close this gap.

Evidence accumulation models have recently been used to analyze the cognitive mechanisms underlying the uptake of social information in binary discrimination tasks (e.g.,^9–12^) and social systems^19^. These models describe the decision process as a continuous process whereby information accumulates over time. The most popular and successful model of evidence accumulation in decision making is arguably the drift-diffusion model (DDM;^20^), which describes the process of choice between two options (for reviews, see^21,22^). The DDM assumes that an individual starts the evidence accumulation process at a given start point (which may be neutral or biased toward one option) and gradually accumulates evidence (described by a drift toward one of the options) over time. As soon as the evidence state reaches a threshold indicating that sufficient evidence has been gathered, a decision is made. Previous studies employing the DDM have shown decision makers the choices of others either before or alongside the decision stimulus (but see^19^). These have found evidence for both accounts of social information integration. Toelch et al.^9^ found that social information was mainly integrated through an instantaneous shift in evidence. Several other studies, in contrast, found that social information was gradually integrated over time via the drift rate^10–12,19^. These latter findings suggest that the presentation time of social information matters because its influence amplifies over time. Here, we aim to disentangle the two cognitive processes by presenting social information only after the stimulus has disappeared and varying the time at which the social information arrives.

**Figure 1:**
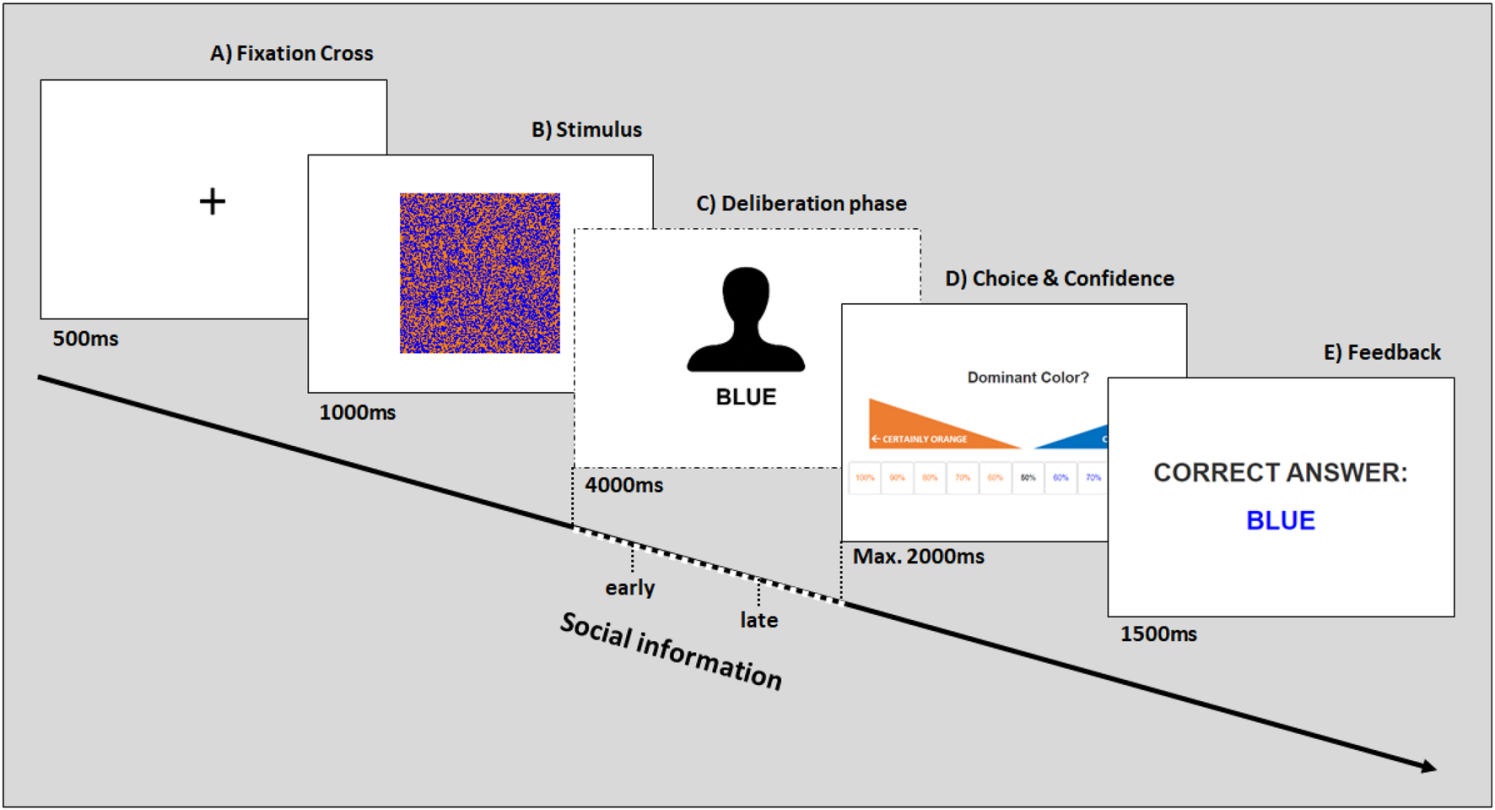
The perceptual decision task used in Experiment 1. Each trial started with (**a**) a fixation cross, followed by (**b**) the stimulus consisting of a square with orange and blue pixels. The task was to determine the dominant color of the square. (**c**) Social information arrived either 750 ms (and lasted for 3250 ms) or 3250 ms (and lasted for 750 ms) after the onset of the deliberation phase, which lasted for 4000 ms. (**d**) Participants then had 2000 ms to make their judgment on an 11-point scale from ‘100% confidence in orange’ via ‘50%’ to ‘100% confidence in blue’. (**e**) Finally, participants received feedback on the correct answer before moving on to the next trial.

In our studies, we used a binary perceptual decision task that requires participants to judge whether the dominant color of a square is orange or blue (Fig. 1). After viewing the stimulus, participants entered a deliberation phase in which they received social information (i.e., the choice of a previous participant) either relatively early or relatively late. After the deliberation phase, they made their judgment, which combined a choice with a confidence judgment. We conducted two experiments. In Experiment 1, early social information was displayed for longer than late social information. In Experiment 2, both early and late social information were shown for the same duration to disentangle the effects of arrival timing and duration of exposure to social information.

**Figure 2:**
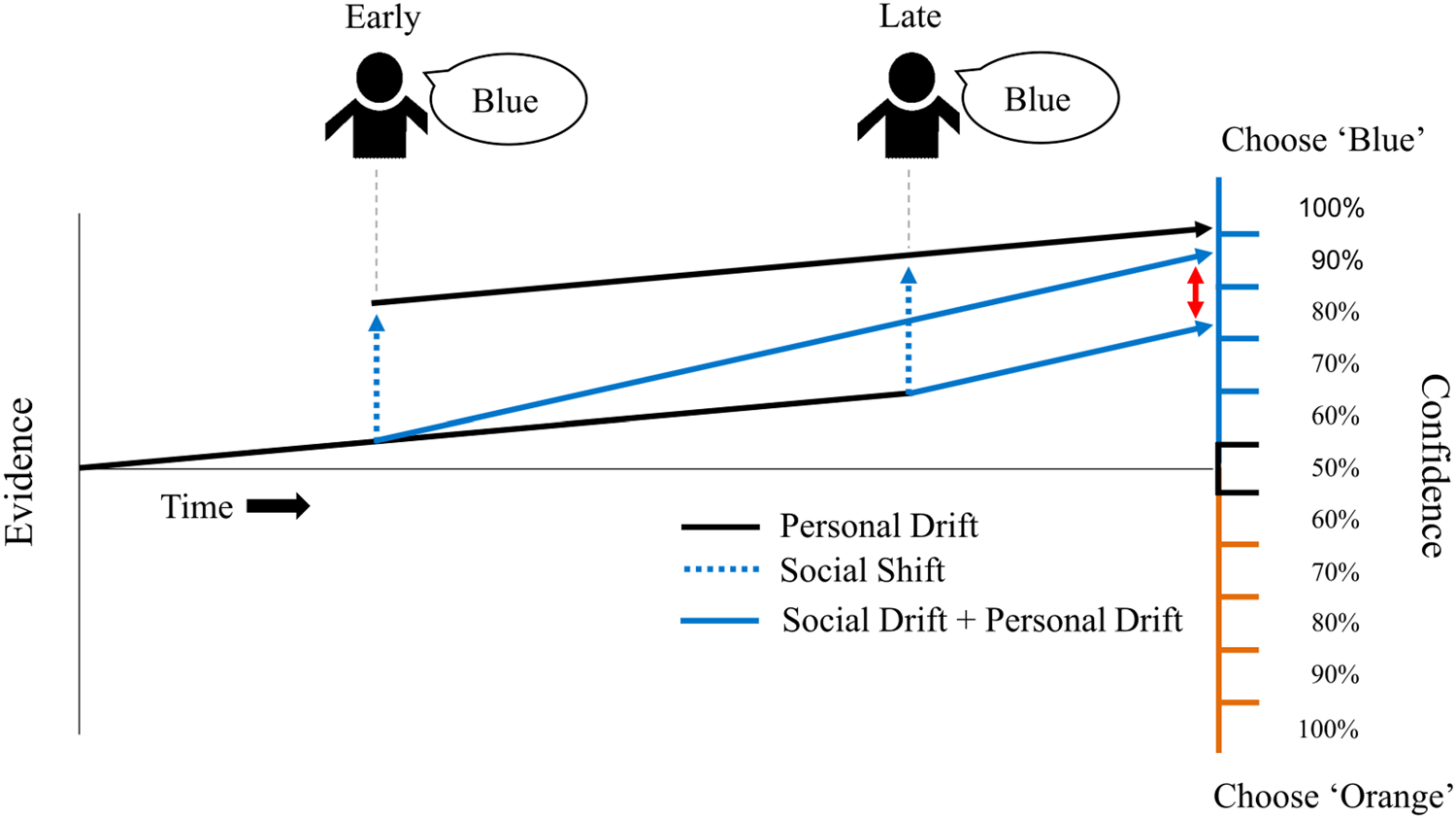
The two processes of social information integration considered in this study. The illustration shows different (stylized) scenarios of evidence accumulation. A decision maker has to judge the dominant color of a stimulus by giving a confidence judgment. At the start, the decision maker’s evidence level is neutral. In this example, the decision maker on average gathers more evidence for the option ‘blue’ (indicated by the increasing solid lines). Social information (i.e., the choice made by a previous participant) arrives either early or late. After the deliberation process, the evidence is mapped onto a confidence judgment. The decision maker can integrate the social information by either (i) instantaneously updating the evidence level toward the option indicated by the social source (i.e., a shift toward ‘blue’; blue dashed arrows) or (ii) biasing the drift toward the option indicated by the social source (blue solid arrows). The two processes of social information integration make different predictions about whether the timing of social information influences the judgment. Earlier social information is expected to have a stronger influence than later information when social information is integrated via a drift because its influence increases over time (indicated by the red arrows). However, social information is expected to have a similar influence when it is integrated via a shift.

As several studies found social information to enter the evidence accumulation process gradually over time (i.e., via a drift;^10–12^), we expected that early social information would have a stronger impact than late social information (see Fig. 2). We expected this to be the case for both correct and wrong social information. Additionally, following findings of a confirmation bias in perceptual decision making^23–26^ and of egocentric discounting in advice taking^13,15,27^, we expected that social information congruent with the stimulus would have a stronger influence than incongruent social information. To disentangle the two described cognitive mechanisms and quantify their relative contribution, we fitted several evidence accumulation models to our data (see also the preregistration of Experiment 2 at https://osf.io/fvbng/).

## Experiment 1

### Methods

For Experiment 1, which was conducted in 2020, we recruited 99 participants online (female: 45, male: 54; mean age ± standard deviation (*SD*): 38.0 ± 10.6 years), using Amazon Mechanical Turk (MTurk). The sample size was informed by^12^, in which the influence of social norms was investigated using very similar perceptual stimuli. Only individuals within the United States, with an MTurk approval rate exceeding 95% and who signed an informed consent form were allowed to participate. All procedures of this study were approved by the Institutional Review Board of the Max Planck Institute for Human Development and were conducted in accordance with relevant guidelines and regulations. Participants performed a perceptual decision task in which they had to determine the dominant color of bi-colored squares. Task and stimuli were adopted from^10–12^ and^28^. Figure 1 provides an overview of a trial with social information (control trials are described below; see https://osf.io/fvbng/ for screenshots of instructions and an example trial). Each trial started with a fixation cross, shown for 500 ms (Fig. 1a), followed by the stimulus, shown for 1000 ms (Fig. 1b). Participants then entered a deliberation phase lasting 4000 ms (Fig. 1c). In this phase, social information arrived either relatively early (on average, 750 ms after the onset of the deliberation phase) or relatively late (on average, after 3250 ms). To maintain high levels of attention, we added uniform distributed random noise to the presentation time of the social information (± 250 ms). Social information was presented in the form of a gender-neutral silhouette labeled with one of the colors (e.g., ‘BLUE’). Participants were informed that the social information was the real color choice of another MTurker for that particular stimulus collected in a pilot study in which participants performed the experiment without social information. This allowed us to conduct the experiment without using deception while keeping a high degree of control over the observed social information. After the deliberation phase, participants were prompted to indicate their judgment—a single decision indicating both choice and confidence—per mouse click on an 11-point scale from ‘100% confidence in orange’ via ‘50%’ to ‘100% confidence in blue’ (Fig. 1D). Participants had 2000 ms to make a judgment. If they exceeded this limit, they still needed to make a judgment before being allowed to proceed to the next screen; however, their judgment did not contribute to their bonus payment (described below). Once participants had made their judgment, the correct answer was displayed for 1500 ms (Fig. 1E) before the next trial started. After every 20 trials, participants could take a self-paced break.

Each participant completed a total of 120 trials across six different treatments (i.e., within-subjects design). Four treatments involved social information in which we manipulated accuracy and timing: early-correct, early-wrong, late-correct, and late-wrong social information. To ensure that the social information provided was, on average, correct (and thus facilitate social information uptake), the treatments with correct social information consisted of 28 trials each, whereas the treatments with incorrect social information consisted of 12 trials each. The average accuracy of social information was therefore 70%. This approximates the performance of individuals tested without social information in a pilot study; it meant that the social information was generally useful, but not always accurate. The accuracy of social information was kept the same across timing conditions (early/late) to decouple the timing of social information from its quality (e.g.,^19,29^). In the fifth treatment, no social information was provided. The sixth treatment involved ‘filler’ trials, which likewise did not contain social information but in which the deliberation phase was only 1000 ms. The ‘filler’ trials were included to keep participants attentive (but were not included in the analysis). In order to ensure that treatments were approximately evenly distributed across the experiment, the 120 trials were grouped into 20 blocks of six trials, with each block containing two trials with early social information, two with late social information, one with no social information, and one filler trial in a randomized order.

As stimuli, we created 120 unique squares, each consisting of 128 $\times$ 128 orange and blue pixels with a random dispersion of color (Fig. 1b) using Matlab (https://www.mathworks.com/). We implemented two difficulty levels: easy and hard. In easy (hard) trials, the dominant color made up 51.5% (50.5%) of the square’s pixels. Stimulus difficulty (easy/hard) and dominant color (orange/blue) were balanced across treatments (e.g., the 20 filler trials consisted of five trials of each of the four combinations of difficulty level and dominant color). The experiment was programmed in LIONESS Lab^30^.

Participants’ bonus payments depended on their judgments, taking into account both choice and confidence. They received points according to a scoring rule based on the Brier score^31^:

1 $$\begin{aligned} points = 100 \times [1-(correct_i-conf_i)^2] - 75 \end{aligned}$$

where $$correct_{i}$$ equaled 1 (0) if the choice on trial *i* was correct (wrong) and where $$conf_{i}$$ was the confidence judgment given on that trial (i.e., .50, .60, ..., 1.00). This scoring rule meant that participants could maximize their earnings by being as accurate as possible while reporting a confidence which matches their actual probability of being correct. To familiarize participants with the scoring scheme, we showed them a table listing the number of bonus points for each combination of correct/wrong choice and confidence level during the instruction period (Fig. S1). Participants who completed the experiment received a participation fee of $4. They received an additional $0.2 for every 100 points earned, with a guaranteed bonus of $1 if they scored less than 500 points.

Of the 99 participants who completed the experiment, 25 were excluded. Participants were excluded for the following reasons: 100% confidence in more than 90% of trials (*n* = 10); average accuracy below 50% (*n* = 2); more than 10 late responses (> 2 s; *n* = 11); higher average confidence when wrong than when correct (*n* = 20); reported not having seen all squares (i.e., technical glitch; *n* = 3). Note that several participants who were excluded fell into multiple categories. The final sample of 74 participants (female: 32, male: 42; mean age ± SD: 37.2 ± 10.2 years) received a mean bonus payment of $1.35, resulting in an hourly wage of approx. $10.

#### Regression analysis

For statistical data analysis, we used Bayesian hierarchical generalized linear models with the ‘brms’ package^32^ in R^33^. We first investigated accuracy as a binary response variable (correct/wrong) using probit regression models, excluding indecisive responses with 50% confidence (see Fig. S2 for the proportion of 50% confidence across social information conditions). We tested how (1) validity of the social information (correct/wrong), (2) stimulus difficulty (easy/hard), (3) stimulus color (orange/blue), and (4) trial number (z-score normalized) affected accuracy. In a second step, we ran a similar model, but replacing validity of social information with social information presence (absent/present) to estimate whether social information had an overall positive effect on accuracy or not.

To analyze the impact of the timing of social information on participants’ judgments, the responses were treated as an ordinal variable with 11 levels ranging from 100% confidence in orange to 100% confidence in blue in 10% increments. We used the ‘brms’ package to fit this as an ordinal variable using ordered probit regression models^34^. Ordered probit regression models assume that the response categories have an order (e.g., a confidence level of 70% is higher than 60%) but that the psychological distance between them is unknown and, therefore, has to be estimated. Using a probit-link function allowed us to perform a signal detection analysis with a regression model^35^. We, thereby, estimated how stimulus difficulty, trial number, and validity of the social information in interaction with its arrival time changes the discrimination ability ($d'$). We combined arrival timing and validity of social information in a single predictor (absent/early-correct/late-correct/early-wrong/late-wrong). Creating a predictor with five levels that captures the interaction of timing and validity of social information external to the regression model helped to disentangle the effects of the two variables. In addition, it avoided parameter estimates of nonsensical interactions between the different levels of timing and validity (e.g., timing: early * validity: absent). We further included individual identity as a random intercept in all the above-mentioned models.

The Bayesian model analyses were conducted by running five parallel Markov Chain Monte Carlo (MCMC) simulations with 5000 iterations which is more than the default setting and enough to get reliable posterior estimates. The first 2500 iterations were discarded as burn-in. In all models, individual identity was included as a group-level effect (i.e., random intercept). As a statistical summary of the regression analyses, we report the means of the posterior distributions and the 95% credible intervals (CI). For all effects for which we had a directional hypothesis (i.e., a predicted direction), we additionally report the Evidence Ratio (ER) as the relative evidence for a positive or negative effect over the alternative, annotated as $ER_+$ or $ER_-$, respectively. The ER was calculated as the ratio of posterior samples compatible with our hypothesized direction to those incompatible with it. Conventionally, ERs between 1 and 3.2 indicate weak evidence for the alternative hypothesis, values between 3.2 and 20 substantial evidence, and values above 20 strong evidence^36^. ERs below 1 indicate evidence for the opposing hypothesis. The Gelman–Ruben statistic (Rhat) and visual inspection of the Markov chains confirmed that all chains converged.

#### Cognitive model analysis

We developed a cognitive model to investigate the potential cognitive processes underlying participants’ judgments. We assumed that personal (i.e., evidence from the presented stimulus) and social information in the form of noisy evidence is accumulated over time and then mapped into a confidence judgment^37^. Social information can enter the subjective evidence via two distinct processes: either through an a instantaneous shift or steadily over time, as described by a drift (see also Fig. 2). Note that, in contrast to most evidence accumulation models, our implementation does not predict response times but only confidence judgments. This implementation was chosen because participants were prompted to make a judgment at a specific time. The evidence distribution at the time point when confidence is reported, $L_c$, is normally distributed with a mean of:

$$\begin{aligned} E(L_c) = \tau * col + \delta _p * DT +(\delta _s* ST + \gamma ) * \lambda \ \end{aligned}$$

and a variance of:

$$\begin{aligned} var(L_c) = \sigma * DT\ \end{aligned}$$

where the free parameters are the uptake of personal information per second denoted by $\delta _p$, the uptake of social information per second denoted by $\delta _s$, the instantaneous integration of social information denoted by $\gamma$, and an initial tendency toward either of the colors, denoted by $\tau$, with positive/negative values indicating a bias toward orange/blue. *DT*, *ST*, *col*, and $\lambda$ are independent variables, with *DT* describing the time between stimulus onset and judgment, *ST* the time between onset of social information and judgment, *col* the dominant color of the stimulus, and $\lambda$ the validity of social information. $\sigma$ describes the diffusion process and scales the free parameters. It is set to 1, as is standard practice in accumulation models^22^.

This evidence space $L_c$ is divided into 11 confidence judgments $conf_j$ with $j = 1, 2,\ldots 11$ by 10 confidence criteria $c_k$ (see also Fig. 2). The probability of reporting confidence judgment $conf_j$ is given by the cumulative normal distribution $N(E[L_c]; var[L_c])$, with $P( -\infty< L_c < c_j )$ for $j=1$, $P( c_{j-1}< L_c < c_j )$ for $j=\{2:10\}$, and $P( c_{j-1}< L_c < \infty )$ for $j=11$. We assume that the confidence criteria are symmetrical for correct and wrong judgments (i.e., $c_5 = -c_6, c_4 = -c_7$, etc.), implying that the psychological space for a specific confidence judgment (e.g., 70%) is the same for correct and wrong judgments. Table 1 provides descriptions of all of the model’s latent free parameters (i.e., the parameters to be estimated by the model) and of the independent variables.

**Table 1:** Features of the cognitive model.

Variable type	Feature	Parameter	Description
Independent variables	Decision time	*DT*	Time between stimulus onset and confidence judgment (in seconds)
	Social influence time	*ST*	Time between onset of social information and confidence judgment (in seconds)
	Color	*col*	Dominant color of the stimulus, with 1/$-1$ indicating orange/blue
	Social information validity	$\lambda$	Validity of social information, with 1/$-1$ indicating correct/wrong
Free parameters	Personal drift	$$\delta _p= {\left\{ \begin{array}{ll} \delta _{easy}, &{} \text {for easy trials}\\ \delta _{hard}, &{} \text {for hard trials}\\ \end{array}\right. }$$	Average uptake of correct evidence from the presented stimulus per second
	Social drift	$\delta _s$	Average uptake of evidence for the option indicated by the social information per second (starting at the onset of the social information)
	Social shift	$\gamma$	Instantaneous shift in accumulated evidence toward the option indicated by the social information
	Color bias	$\tau$	Initial tendency to favour one of the colors, with positive/negative values indicating orange/blue
	Confidence criteria	$c_k$	Thresholds that divide the evidence space into confidence judgments

For the cognitive model analysis, we used the same exclusion criteria as for the regression analysis, and additionally excluded trials in which participants did not respond in the allocated time ($\approx 5\%$ in Experiment 1; $\approx 3\%$ in Experiment 2). The analysis was conducted using *RStan*^38^, with an hierarchical framework to control for subject-level differences. We ran five MCMC chains in parallel with 5000 iterations each, discarding the first 2500 as burn-in. The main parameters of interest are the personal drift ($\delta _p$), social drift ($\delta _s$), and social shift ($\gamma$). We fitted models with all possible combinations of these parameters (i.e., eight models) and identified the model best predicting the data using the Leave-one-out information criterion (LOOIC). LOOIC is an information criterion for predicted (out-of-sample) accuracy and widely used for model comparisons^39^. We report the means, 95% CI and Evidence Ratios ($ER_{+/-}$), but only for effects for which we had a directional hypothesis, as these are one-sided tests. We verified the validity of the model by conducting a parameter recovery analysis (see Fig. S3). For all parameters, the input and recovered parameters were strongly aligned and highly correlated, confirming the interpretability of the parameter estimates in relative and absolute magnitudes. The Gelman–Ruben statistic (Rhat) and visual inspection of the Markov chains confirmed that all chains converged.

### Results

**Figure 3:**
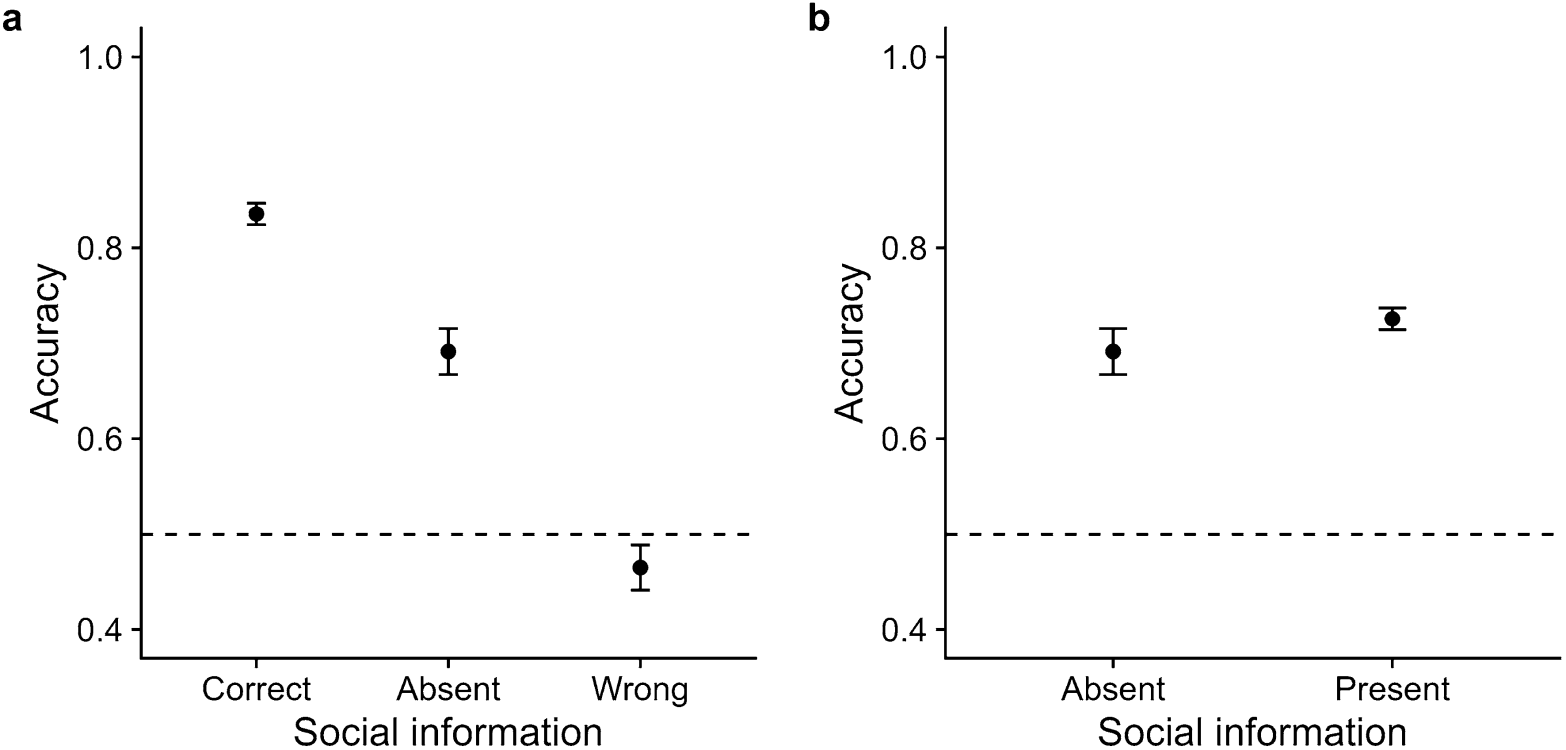
Accuracy as a function of social information in Experiment 1. Accuracy (**a**) by the validity of social information and (**b**) in the absence/presence of social information. Dots indicate raw means; error bars indicate twice the standard error. Horizontal dashed lines indicate chance performance.

Participants achieved an average accuracy of 69.1% in trials without social information (Fig. 3a). Accuracy increased when participants received correct social information (mean accuracy = 83.6%, $\beta _{SI-correct} = 0.52$, CI = [0.42, 0.59], $ER_+$ > 100) but reduced when they received wrong social information (mean accuracy = 46.5%, $\beta _{SI-wrong} = -\,0.61$, CI = [−0.70, − 0.52], $ER_-$ > 100), showing that participants incorporated the social information. Overall, participants achieved slightly higher accuracy in trials with social information (mean accuracy = 72.6%) than in trials without ($\beta _{SI} = 0.11$, CI = [0.03, 0.19]; Fig. 3b), indicating a slight net benefit from social information use. Figure S5 shows the influence of stimulus difficulty, stimulus color, and trial number on accuracy and Table S1 presents the regression model summary.

**Figure 4:**
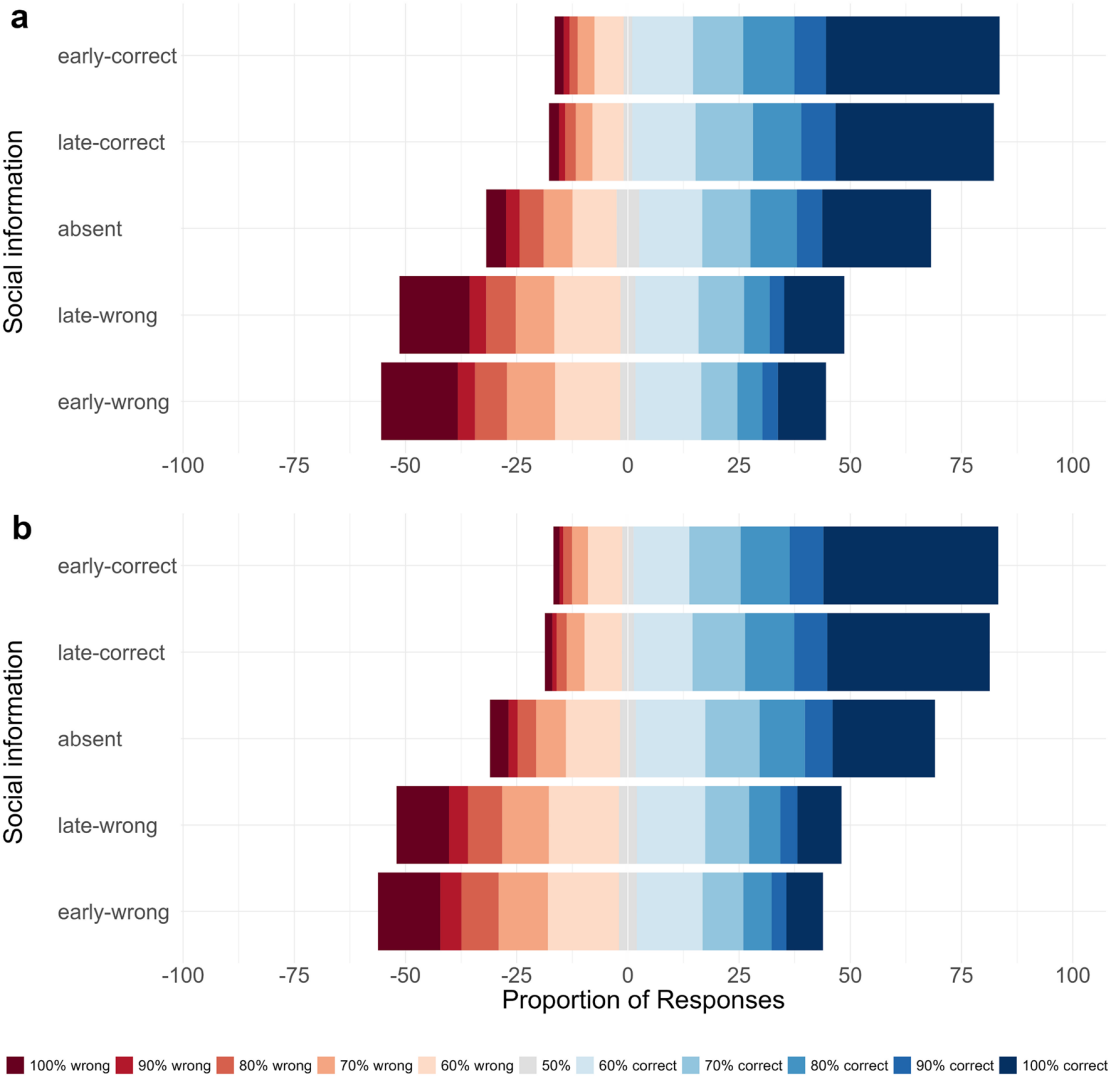
Distribution of confidence judgments in Experiment 1. Proportion of confidence judgments by treatment, (**a**) as observed in the experiment and (**b**) as predicted by the Bayesian regression model (see Table S2). Blue/red colors show proportions of confidence judgments for correct/wrong choices. Gray indicates neutral choices (i.e., 50%). Note the relatively high proportion of trials with the highest confidence levels (darkest shades of blue/red). Although this model provided a good representation of the empirical results, it slightly underestimated the number of wrong choices given with the highest confidence (100%).

Next, we investigated the influence of social information on choice accuracy in combination with confidence judgments using a signal detection analysis. Figure 4 shows the proportion of judgments in each of the 11 confidence categories (from 100% confident and wrong, via 50% confident, to 100% confident and correct) by treatment for the empirical data and the model predictions (see Table S2 for model summaries and S4 for the influence of social information on average confidence). In line with the results above, we find that in the absence of social information participants have a discrimination ability well above zero ($d' = 1.43$, CI = [1.31, 1.55], $ER_+$ > 100). Both early-correct and late-correct social information substantially improved participants’ discrimination ability ($\Delta d'_{early-correct} = 0.95$, CI = [0.80, 1.09], $ER_+$ > 100; $\Delta _{late-correct} = 0.80$, CI = [0.65, 0.94], $ER_+$ > 100). Likewise, both early-wrong and late-wrong social information were detrimental to participants’ performance ($\Delta d'_{early-wrong} = -\,1.32$, CI = [−1.50, −1.14], $ER_-$ > 100; $\Delta d'_{late-wrong} = -\,1.10$, CI = [−1.28, −0.94], $ER_{-}$ > 100). Comparing the effect sizes for social information between the two valence categories of social information showed that, contrary to our expectation, wrong social information had a larger influence on participants’ judgments than correct social information. This held true for both early and late social information ($|\Delta d'_{early-correct}| - |\Delta d'_{early-wrong}| = -0.37$, CI = [$-0.65, -0.10$], $ER_{+} < 1/100$; $|\Delta d'_{late-correct}| - |\Delta d'_{late-wrong}| = -0.31$, CI = [$-0.58, -0.04$], $ER_{+} < 1/100$). Note that ERs smaller than 1/20 indicate strong evidence for effect sizes opposing our initial hypothesis. We elaborate on this finding in the discussion. Participants’ discrimination ability was poorer in hard trials ($\Delta d'_{Hard} = -0.85$, CI = [$-0.95, -0.75$]), and increased slightly over time ($\Delta d'_{Trial} = 0.14$, CI = [0.10, 0.19]).

When investigating the influence of timing, we found that the positive effect of correct social information was larger for early than for late social information ($\Delta d'_{early-correct} - \Delta d'_{late-correct} = 0.15$, CI = [0.02, 0.28], $ER_+$ = 71). Likewise, the negative effect of wrong social information was larger for early than for late social information ($\Delta d'_{early-wrong} - \Delta d'_{late-wrong} = -0.21$, CI = [−0.40, −0.02], $ER_{-}$ = 62). Thus, early social information had a stronger influence on participants’ judgments compared to late social information and this effect was irrespective of its accuracy ($(\Delta d'_{early-correct} - \Delta d'_{late-correct}) - |(\Delta d'_{early-wrong} - \Delta d'_{late-wrong})| = -0.06$, CI = [-0.26, 0.13]). The signal detection model was able to recover the confidence distributions, the positive/negative influence of correct/wrong information, and the stronger effect of early social information (Fig. 4B). It also recovered the relationship between confidence and accuracy across all social information conditions (Fig. S6).

To investigate the cognitive mechanisms underlying these results, we fitted the evidence accumulation model to the data. The best model (i.e., lowest LOOIC) included all three main parameters of interest: personal drift, social drift, and social shift (see Table S3 and S4). On average, participants extracted evidence for the correct option from observing the stimulus, as described by a positive personal drift ($\delta {easy} = 0.90$, CI = [0.81, 0.99], $ER_{+}$ > 100). Social information was integrated via two distinct processes. First, participants drifted toward the option indicated by the social information ($\delta {s} = 0.18$, CI = [0.06, 0.30], $ER_{+}$ > 100). This explains why early social information had a stronger influence (see also Fig. 2). Second, participants’ level of evidence made an instantaneous shift toward the option indicated by the social information ($\gamma = 2.41$, CI = [1.92, 2.91], $ER_{+}$ > 100). Both processes, the social drift and shift were thereby simultaneously present in a large majority of subjects (Fig. S7). The effect size of the social drift describes a change in evidence space per second (see also Fig. 2). With judgments being made on average 3.1 seconds after the onset of the social information, the total adjustment in evidence space via the social drift was approximately 0.56. The adjustment via an instantaneous shift was approximately 2.41 and thus roughly four times as large. As the confidence criteria were roughly 1.5–2 units of evidence space apart (see Table S4) both together result in a confidence change of approximately 10%. Importantly, and similar to the signal detection model, the evidence accumulation model was able to recover the confidence distributions, the influence of correct and wrong information, and the stronger effect of early social information (Fig. S8).

## Experiment 2

### Methods

In Experiment 1, all social information was displayed until the end of the deliberation phase, meaning that early information was displayed for longer. Experiment 2 aimed to disentangle the possible effects of arrival timing and display duration. Social information was again provided either early or late (750 ms or 3250 ms after the onset of the deliberation phase), but in both conditions it was only displayed for 500 ms, after which it disappeared. The experimental design was otherwise as in Experiment 1, with two further changes. First, during the feedback stage, we displayed the number of points won/lost in addition to the correct answer. This was done because participants in Experiment 1 relatively often used the highest confidence category (i.e., 100%; see Fig. 4). We aimed to make the (nonlinear) scoring rule (see Fig. S1) more salient by showing the points gained or lost during the feedback stage. Second, participants had 3 seconds (instead of 2 seconds) to make their judgment. This change was made to reduce the number of excluded judgments.

Experiment 2 was preregistered (https://osf.io/fvbng). As part of the preregistration, we conducted a power analysis to estimate the number of participants needed to reliably detect a credible effect of timing (i.e., stronger influence of earlier correct/wrong social information) based on the effect sizes in Experiment 1. The power analysis yielded a target sample size of 200 to achieve a power of 0.8 for both effects. For Experiment 2, which was conducted in 2021, we recruited 282 participants, of whom 79 (28%) were excluded based on the same exclusion criteria as in Experiment 1. This resulted in a final sample of 203 participants (female: 69, male: 133, nonbinary: 1; mean age ± SD: 39.33 ± 11.38 years). Participants received a mean bonus payment of $1.15 and a participation fee of $4, resulting in the equivalent of an hourly wage of approx. $10.

### Results

**Figure 5:**
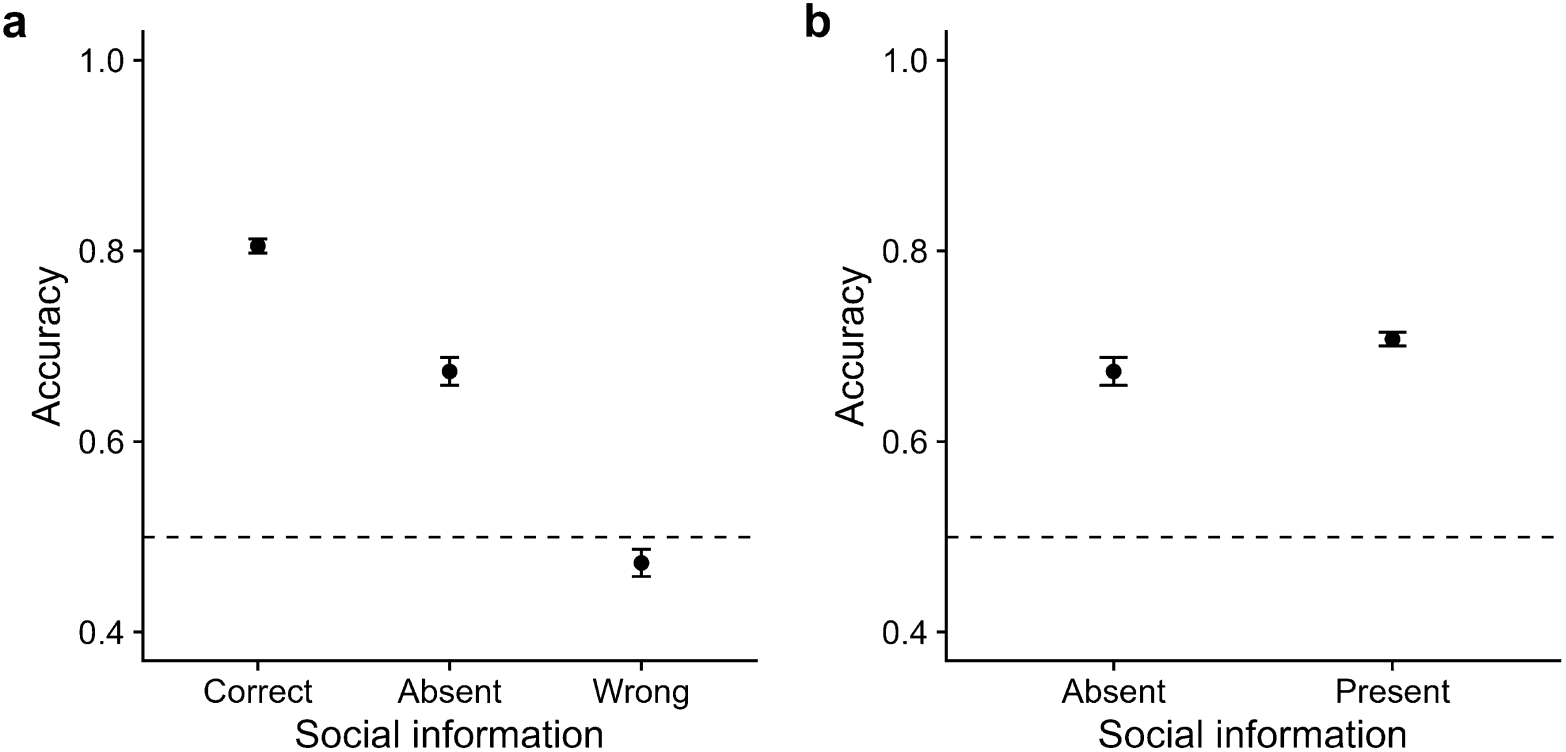
Accuracy as a function of social information in Experiment 2. Accuracy (**a**) by validity of social information and (**b**) in the absence/presence of social information. Dots indicate means; error bars indicate twice the standard error. Horizontal dashed lines indicate chance performance.

The effect of social information on judgments was largely identical to that observed in Experiment 1. Participants achieved an average accuracy of 67.3% in trials without social information (Fig. 5a). They used the displayed social information as indicated by the accuracy improvement with correct social information (mean accuracy = 80.5%, $\beta _{SI-correct} = 0.43, CI = [0.38, 0.48]$, $ER_{+}$ > 100), and accuracy deterioration with wrong social information (mean accuracy = 47.2%, $\beta _{SI-wrong} = -0.54$, CI = [$-0.60, -0.48$], $ER_{-}$ > 100). Overall, participants’ accuracy increased with social information (mean accuracy = 70.7%, $\beta _{SI} = 0.10$, CI = [0.05, 0.15]; Fig. 5b). Also, the effects of stimulus difficulty, stimulus color, and trial number on accuracy remain similar in Experiment 2 (see Fig. S9; and Table S5 for the regression model summary).

**Figure 6:**
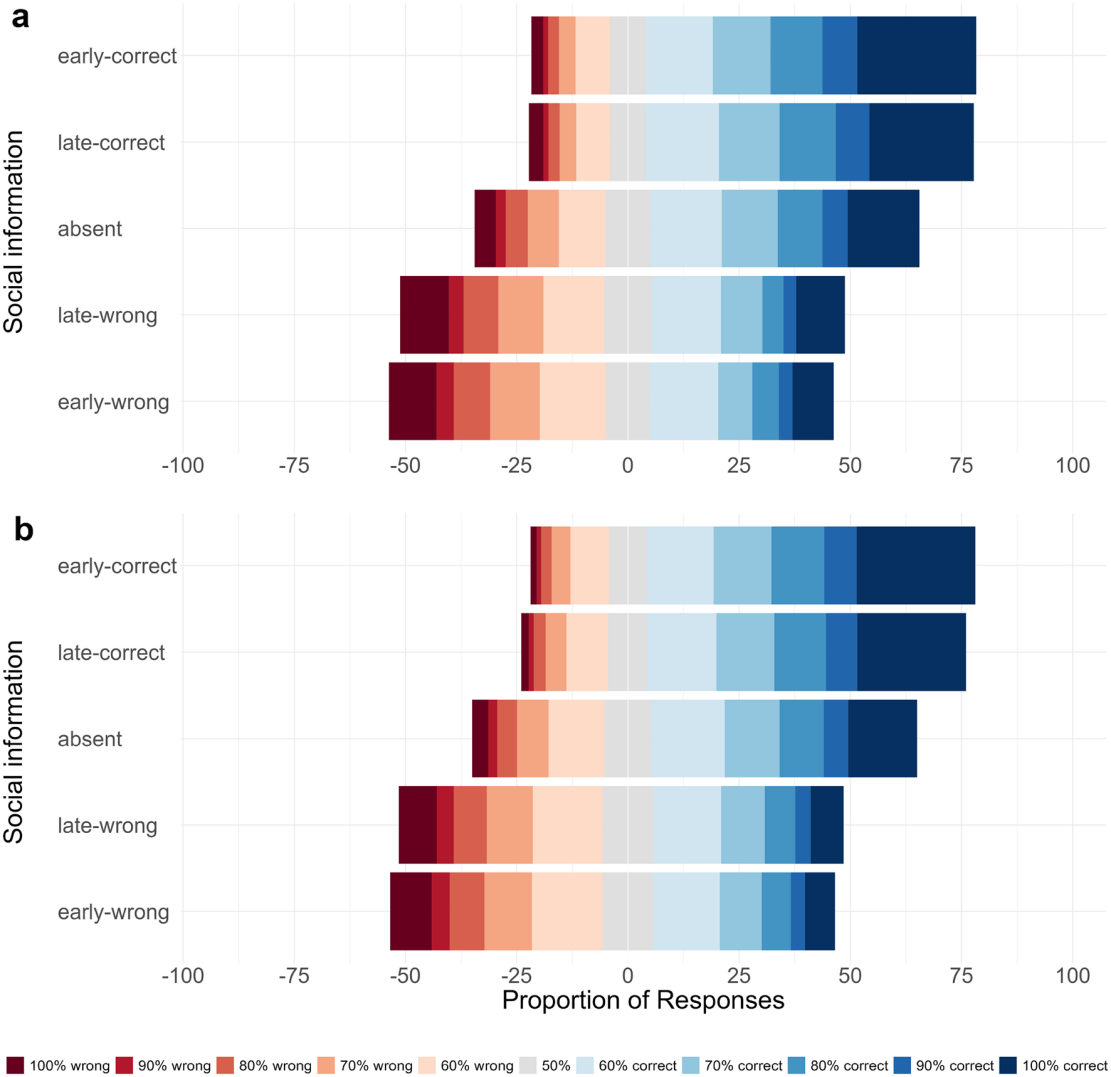
Distribution of confidence judgments in Experiment 2. Proportions of confidence judgments by treatment, (**a**) as observed in the experiment and (**b**) as predicted by the Bayesian regression model (see Table S6). Blue/red colors show proportions of confidence judgments for correct/wrong choices. Gray indicates neutral choices (i.e., 50%). Note the relatively high proportion of trials with the highest confidence levels (darkest shades of blue/red). The model recovers the overall empirical patterns well.

Both early and late correct social information improved participants’ performance ($\Delta d'_{early-correct} = 0.80$, CI = [0.71, 0.88], $ER_{+}$ > 100; $\Delta d'_{late-correct} = 0.66$, CI = [0.57, 0.74], $ER_{+}$ > 100), with early information having a larger positive effect ($\Delta d'_{early-correct} - \Delta d'_{late-correct} = 0.14$, CI = [0.08, 0.21], $ER_{+}$ > 100; Fig. 6a). Likewise, both early and late wrong social information was detrimental to participants’ performance ($\Delta d'_{early-wrong} = -0.96$, CI = [$-1.06, -0.86$], $ER_{-}$ > 100; $\Delta d'_{late-wrong} = -0.86$, CI = [$-0.97, -0.76$], $ER_{-}$ > 100), with early information having a larger negative effect ($\Delta d'_{early-wrong} - \Delta d'_{late-wrong} = -0.10$, CI = [−0.21, 0.01], $ER_{-}$ = 19.5). The Evidence Ratio indicated strong evidence for this effect, although the credible interval slightly overlapped zero. As in Experiment 1, the timing effect is equally pronounced for both correct and wrong information ($(\Delta d'_{early-correct} - \Delta d'_{late-correct}) - |(\Delta d'_{early-wrong} - \Delta d'_{late-wrong})| = 0.07$, CI = [-0.07, 0.16]). Taken together, Experiment 2 further corroborates the importance of the timing of social information. Early social information has a stronger influence on participants’ judgments than late social information, even when both are displayed for the same duration.

Consistent with Experiment 1, wrong social information had a larger influence on participants’ judgments than correct social information. This held for both early and late social information ($|\Delta d'_{early-correct}| - |\Delta d'_{early-wrong}| = -0.17$, CI = [$-0.32, -0.01$], $ER_{-}$ = 1/50; $|\Delta d'_{late-correct}| - |\Delta d'_{late-wrong}| = -0.21$, CI = [$-0.37, -0.05$], $ER_{+} < 1/100$). See Table S6 for model summaries. The models can recover all important empirical patterns (see Fig. 6b), including the confidence-accuracy relationship (see Fig. S10).

Again, we investigated the underlying cognitive mechanisms by fitting an evidence accumulation model and found that—consistent with the results of Experiment 1—the best model included all three main parameters (see Table S3, Table S4, and Fig. S12). On average, participants gathered evidence for the correct option from the stimulus, as described by a positive personal drift ($\delta {easy} = 0.81$, CI = [0.75, 0.87], $ER_{+}$ > 100). Social information was integrated via two distinct processes: a continuous drift ($\delta {s} = 0.14$, CI = [0.07, 0.21], $ER_{+}$ > 100) and an instantaneous shift ($\gamma = 2.06$, CI = [1.77, 2.36], $ER_{+}$ > 100), both toward the option indicated by the social information. Both processes were again simultaneously present in a large majority of subjects (Fig. S11). With judgments being made on average 3.3 seconds after the onset of the social information, the total adjustment in evidence space via the social drift was approx. 0.46. The adjustment via an instantaneous shift was approx. 2.06 and hence about four times as large (for model predictions, see Fig. S12). An adjustment of two in evidence space thereby corresponds to a confidence change of about 10% (see Table S4).

## Discussion

Our results emphasize the importance of the timing of social information for its subsequent uptake. Independent of whether it was correct or wrong, earlier social information had a stronger effect on participants’ judgments. Thus, early received social information had a larger impact and this finding persisted when we controlled for the display length of the social information (in Experiment 2). Using an evidence accumulation approach, we found that the integration of social information was best explained by two separate processes: an instantaneous shift and a continuous drift toward the option indicated by the social source, with the former having a stronger effect on judgments.

The social drift implies that the presence of social information continuously biases the evidence accumulation process toward the option indicated by the social source. This could be explained by several non-mutually exclusive cognitive mechanisms. First, the social information itself could receive increasing weight over time. A similar process has been described in other choice domains, where longer attention to an option feature can increase the value of the option^40,41^. Second, social information incorporation could include a careful and lengthy deliberation process that comprises the evaluation of several social attributes (e.g., believed accuracy, social norms, reputation;^42^) and, therefore, unfolds its influence over time. Third, the presence of social information could bias an individual’s deliberation on perceptual information toward the option favoured by the social source^10–12^. Previous studies have shown that individuals preferentially collect information^43,44^ or arrive at conclusions that support the social source (i.e., motivated reasoning;^45,46^ and confirmation bias, see below). In our case, it could be that participants draw more evidence (about the stimulus) from memory that aligns with the social source. When social information is incorporated in an instantaneous social shift, in contrast, the effect does not strengthen over time; this process describes how rational (Bayesian) individuals are expected to incorporate evidence: by a one-time update (i.e., a shift) of the evidence level^7,8,47^.

Previous studies have investigated how social information enters the evidence accumulation process when it appears either before or during the stimulus presentation. Their results are mixed, with evidence emerging both for continuous integration of social information over time (i.e., a bias in the uptake of available perceptual information;^10,11^) and for a one-time integration^9^. This discrepancy has been explained by the type of social influence: normative versus informational. Some of the studies provided social information that did not have informational value (i.e., its accuracy was no better than chance). These studies found that the normative influence unfolded its impact over time and was best described by a change in the drift rate^10,11^. When the observed choices had informational value, in contrast, choice behavior was best explained by a one-time integration^9^. In our study, the effect of the timing of social information was small and most of the social impact was explained by a one-time integration. As the social information in our study predominantly indicated the correct option, our results are in line with these earlier findings suggesting that social input with informational value is more likely to be updated via a shift. But why would informational and normative influences differ in how they unfold over time? Although a one-time update would be expected by a rational agent, simple heuristic strategies (in contrast to lengthy deliberation processes), also predict a quick incorporation of ‘simple’ evidence (see e.g., mere consensus;^48^). In contrast, a drift describes a more lengthy deliberation process after observing the social source. The co-occurrence of two such distinct cognitive processes is predicted by dual-process models which often distinguish between fast heuristic and slow deliberate processes (see e.g.,^42,49^). Thus, individuals could rely on different strategies depending on whether they are confronted with social influence in an informational^9^ versus social norm context^10,11^.

Whereas in these previous studies, social information typically appeared before or during the stimulus presentation, in our experiments it appeared after the stimulus presentation. Both situations may arise in the real world (e.g., receiving a restaurant recommendation from a friend before reading the menu versus reading a description of a consumer product before consulting the reviews). These differences in the order of appearance of information may give rise to different cognitive processes. When social information is available before the stimulus, for example, it seems reasonable to predict that the process of social information biasing the information extraction may be stronger.

Although the observed effect of timing can be explained by a continuous integration of social information via a drift, we cannot rule out an alternative explanation, namely stronger shifts when observing early social information. If, for example, participants judge fast choices to be more reliable and therefore update their evidence with a stronger shift when choices are presented earlier, these choices would exert a stronger social influence. It is a common finding that correct choices are made faster than incorrect ones^50,51^. Boundedly rational decision makers are expected to make use of such environmental regularities—for example, by inferring the distance of an object from its size^52^ or the risk (i.e., probability) of a choice from its potential rewards^53^. Indeed, if accurate choices are made faster, and in the absence of other cues, individuals use response times to infer others’ accuracy^29^. Although early and late information had the same information value in our paradigm, participants might have used this heuristic and given slightly more weight to early information.

Confirmation bias—that is, the tendency to interpret information such that it confirms one’s prior beliefs—is a robust finding across many areas of decision making, also in a social context^23–26^. We, therefore, expected social information congruent with the stimulus (i.e., correct social information) to have a stronger influence on judgments than incongruent social information. However, the opposite was the case: Incongruent (or wrong) social information had a stronger impact on judgments than congruent (or correct) information. This finding is most likely an artefact stemming from the fact that participants’ judgments were, on average, correct. In binary choice, an improvement in discrimination ability can be difficult to estimate if accuracy is close to ceiling, while a reduction in discrimination ability toward chance levels exerts stronger accuracy changes^54^. In our experiment, most judgments were on the accurate side of the confidence scale; accordingly, wrong social information can do more harm than correct social information can do good. Our post hoc conclusion is that our paradigm is ill-suited to test how confirmation bias influences belief updating presence of social information. To do so, one would need to be able to access participants’ evolving beliefs during the decision process. This is notoriously difficult but can be approximated with other set-ups, such as mouse-tracking paradigms^55^ or in sampling paradigms where the probabilistic samples (i.e., information) provided over time are known by the experimenter (e.g.,^56^). Future research should investigate the dynamic interplay of held beliefs and incoming social information in more detail; this approach might be especially interesting in contexts where people often exhibit strong confirmation biases, such as the detection of false political news. Confirmation bias has also been linked to the primacy effect^24,57^, an information processing bias found in many judgment and decision making contexts describing the tendency of individuals to assign more weight to information acquired early^58–60^. Both a confirmation bias and a primacy effect could explain the stronger influence of early information if the timing of social information also changed the sequence in which individuals deliberated over evidence. Both effects have been described previously in social (e.g.,^16,61,62^) and non-social contexts. It remains an open question how much the timing effect is influenced by such sequence dependencies in interaction with the (social) nature of the information source.

Future work can extend our paradigm in several ways. First, we only used one piece of social information. Future research could investigate how the presence of multiple pieces of social information appearing at different time points (and in different orders) influences judgments. Differences in response times might be particularly salient when multiple choices are observable over time. If earlier choices indeed have a stronger influence, two opposing choices should still sway judgments in the direction of the faster choice. Second, our experiments took place on a time scale of seconds, and participants had to wait during a deliberation phase set by the experimenter to ensure that they observed the social stimulus even when it arrived late. Future work could investigate whether our results generalize to different time scales, such as minutes, hours, or even longer, and to tasks where individuals are free to time their judgment. Results indicating that the arrival time of social information remains important on the longer term might have important implications for the emergence of opinions in social systems (e.g., the spread of false news online or the emergence of bestseller products).

In conclusion, our results contribute to the growing literature studying how social information is incorporated over time. We found that early social information has a stronger impact on judgments, which is best explained by both an immediate, one-time update of evidence and continuous integration of social information over time. Our findings advance the understanding of how social influence shapes decision-making processes and provide new insights into the temporal evolution of social impact, the propagation of information, and the emergent social dynamics.

### Supplementary Information

Supplementary Information.

## Data Availability

The empirical data and code for the analyses can be accessed at https://osf.io/fvbng/. All measures, manipulations, and exclusions in these studies are disclosed.
