# Supplementary material for: Earlier social information has a stronger influence on judgments
Source: Sci Rep. 2024 Jan 2;14:105. doi: 10.1038/s41598-023-50345-4 (PMC10762246; doi:10.1038/s41598-023-50345-4)
Supplement: Supplementary file 1 — Supplementary Information. [file 41598_2023_50345_MOESM1_ESM.pdf]

Supplementary Information to:
Earlier social information has a stronger influence on judgments

Supplementary Tables

Table S1. Bayesian linear regression results with accuracy as predictor (Exp. 1; SI = social information).

**Response**<br>Predictor	Estimate	l-95% CI	u-95% CI	Rhat
**Accuracy (No SI vs. Correct SI vs. Wrong SI)**				
Intercept	0.90	0.81	0.99	1
Hard	-0.48	-0.54	-0.41	1
Orange	-0.29	-0.35	-0.22	1
Correct SI	0.51	0.42	0.59	1
Wrong SI	-0.61	-0.70	-0.52	1
Trial	0.08	0.04	0.11	1
**Accuracy (Absent SI vs. Present SI)**				
Intercept	0.86	0.77	0.95	1
Hard	-0.43	-0.49	-0.36	1
Orange	-0.26	-0.32	-0.19	1
SI	0.11	0.03	0.19	1
Trial	0.06	0.03	0.09	1

Table S2. Bayesian Signal Detection results analyzing the effect of timing (Exp. 1).

**Response**<br>Predictor	Estimate	l-95% CI	u-95% CI	Rhat
**Confidence (100% Orange - 100% Blue)**				
Criterion [1]	-1.39	-1.47	-1.31	1
Criterion [2]	-1.20	-1.28	-1.12	1
Criterion [3]	-0.92	-1.00	-0.84	1
Criterion [4]	-0.61	-0.69	-0.53	1
Criterion [5]	-0.19	-0.27	-0.12	1
Criterion [6]	-0.09	-0.16	-0.01	1
Criterion [7]	0.31	0.24	0.39	1
Criterion [8]	0.62	0.55	0.70	1
Criterion [9]	0.91	0.83	0.99	1
Criterion [10]	1.11	1.03	1.19	1
d’	1.43	1.31	1.55	1
$\Delta d'$ Early + Correct	0.95	0.80	1.09	1
$\Delta d'$ Early + Wrong	-1.32	-1.50	-1.14	1
$\Delta d'$ Late + Correct	0.80	0.65	0.94	1
$\Delta d'$ Late + Wrong	-1.10	-1.28	-0.93	1
$\Delta d'$ Hard	-0.85	-0.95	-0.75	1
$\Delta d'$ Trial	0.14	0.10	0.19	1

Table S3. Comparison of different models based on LOOIC with different combinations of the main parameters of interest (Exp. 1+2).

Personal drift	Social drift	Social shift	LOOIC (rank) Exp. 1	LOOIC (rank) Exp. 2
not included	not included	not included	27502 (8)	79267 (8)
included	not included	not included	25616 (6)	74418 (6)
not included	included	not included	25982 (4)	75918 (4)
included	included	not included	24345 (2)	71641 (2)
not included	not included	included	25736 (7)	75445 (7)
included	not included	included	24141 (5)	71280 (5)
not included	included	included	25735 (3)	75444 (3)
included	included	included	24136 (1)	71267 (1)

Table S4. Priors and estimates of the group-level parameters of the cognitive model (Exp. 1+2).

Parameters	Priors	Estimates Exp. 1	Estimates Exp. 2
δ_{easy}	N(0, 1)	0.90 [0.81, 0.99]	0.81 [0.75, 0.87]
$\delta_{easy} - \delta_{hard}$	N(0, 1)	-0.49 [-0.55, -0.43]	-0.46 [-0.50, -0.43]
δ_s	N(0, 1)	0.18 [0.06, 0.30]	0.14 [0.07, 0.21]
γ	N(0, 1)	2.41 [1.92, 2.91]	2.06 [1.77, 2.36]
τ	N(0, 1)	-1.45 [-2.19, -0.71]	-1.57 [-2.04, -1.11]
c_6	N(0, 1)	0.29 [0.20, 0.39]	0.84 [0.70, 0.97]
$c_7 - c_6$	N(0, 1)	2.26 [1.82, 2.69]	2.50 [2.23, 2.76]
$c_8 - c_7$	N(0, 1)	1.87 [1.61, 2.14]	2.14 [1.95, 2.34]
$c_9 - c_8$	N(0, 1)	1.82 [1.55, 2.09]	2.11 [1.90, 2.32]
$c_{10} - c_9$	N(0, 1)	1.31 [1.05, 1.59]	1.63 [1.42, 1.86]

Table S5. Bayesian linear regression results with accuracy as predictor (Exp. 2; SI = social information).

**Response**<br>Predictor	Estimate	l-95% CI	u-95% CI	Rhat
**Accuracy (No SI vs. Correct SI vs. Wrong SI)**				
Intercept	0.86	0.81	0.92	1
Hard	-0.48	-0.52	-0.44	1
Orange	-0.30	-0.34	-0.26	1
Correct SI	0.43	0.38	0.48	1
Wrong SI	-0.54	-0.60	-0.48	1
Trial	0.05	0.03	0.07	1
**Accuracy (Absent SI vs. Present SI)**				
Intercept	0.84	0.78	0.90	1
Hard	-0.45	-0.48	-0.41	1
Orange	-0.28	-0.32	-0.24	1
SI	0.10	0.05	0.15	1
Trial	0.05	0.03	0.07	1

Table S6. Bayesian Signal Detection results analyzing the effect of timing (Exp. 2).

**Response**<br>Predictor	Estimate	l-95% CI	u-95% CI	Rhat
**Confidence (100% Orange - 100% Blue)**				
Criterion [1]	-1.55	-1.60	-1.50	1
Criterion [2]	-1.36	-1.40	-1.31	1
Criterion [3]	-1.05	-1.09	-1.00	1
Criterion [4]	-0.72	-0.76	-0.67	1
Criterion [5]	-0.29	-0.33	-0.24	1
Criterion [6]	0.00	-0.04	0.05	1
Criterion [7]	0.41	0.37	0.46	1
Criterion [8]	0.75	0.70	0.80	1
Criterion [9]	1.06	1.01	1.11	1
Criterion [10]	1.29	1.24	1.34	1
d’	1.17	1.10	1.24	1
$\Delta d'$ Early + Correct	0.80	0.71	0.88	1
$\Delta d'$ Early + Wrong	-0.96	-1.06	-0.86	1
$\Delta d'$ Late + Correct	0.66	0.57	0.74	1
$\Delta d'$ Late + Wrong	-0.86	-0.97	-0.76	1
$\Delta d'$ Hard	-0.77	-0.82	-0.71	1
$\Delta d'$ Trial	0.04	0.01	0.07	1

Supplementary Figures

Correct/Wrong	Confidence	Bonus points
Correct	100%	25
Correct	90%	24
Correct	80%	21
Correct	70%	16
Correct	60%	9
Correct/Wrong	50%	0
Wrong	60%	-11
Wrong	70%	-24
Wrong	80%	-39
Wrong	90%	-56
Wrong	100%	-75

Figure S1. Pay-off scheme. This table was shown to participants prior to the experimental trials along with an explanation. The scoring rule based on the Brier score ensured that participants maximized their points (and earnings) when they were as accurate as possible while reporting a confidence that matches their actual probability of being correct.

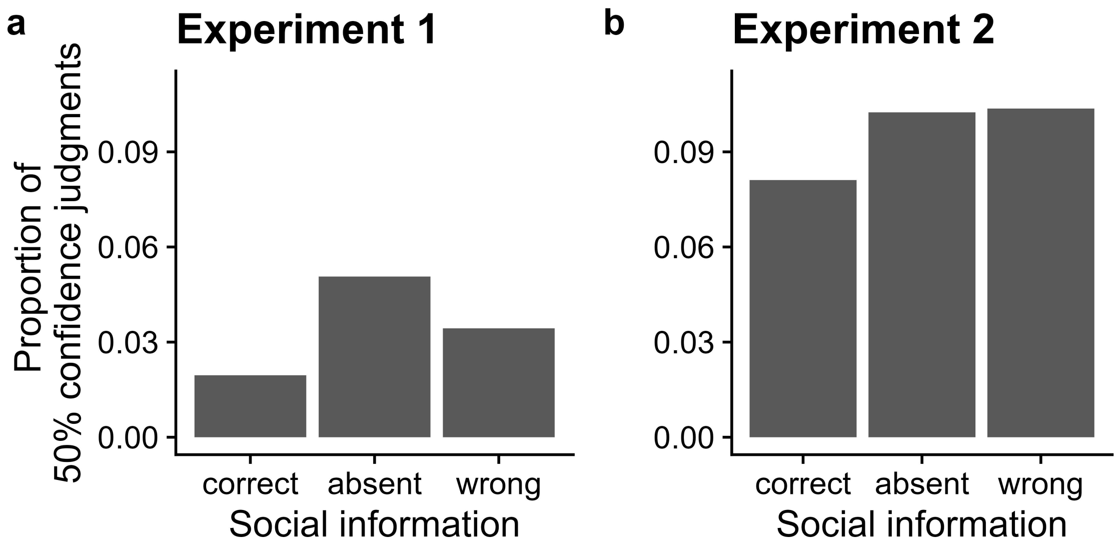

Figure S2. The proportion of 50% judgments across social information conditions for (a) Experiment 1 and (b) Experiment 2. In experiment 1 participants reported a confidence of 50% in approximately 2-5% of the trials while they reported such confidences in 8-10% of the trials in experiment 2. The change in frequency can be explained by the changed feedback stage. In experiment 2, participants observed after each trial the number of points won/lost in addition to the correct answer, emphasizing the danger of being overconfident.

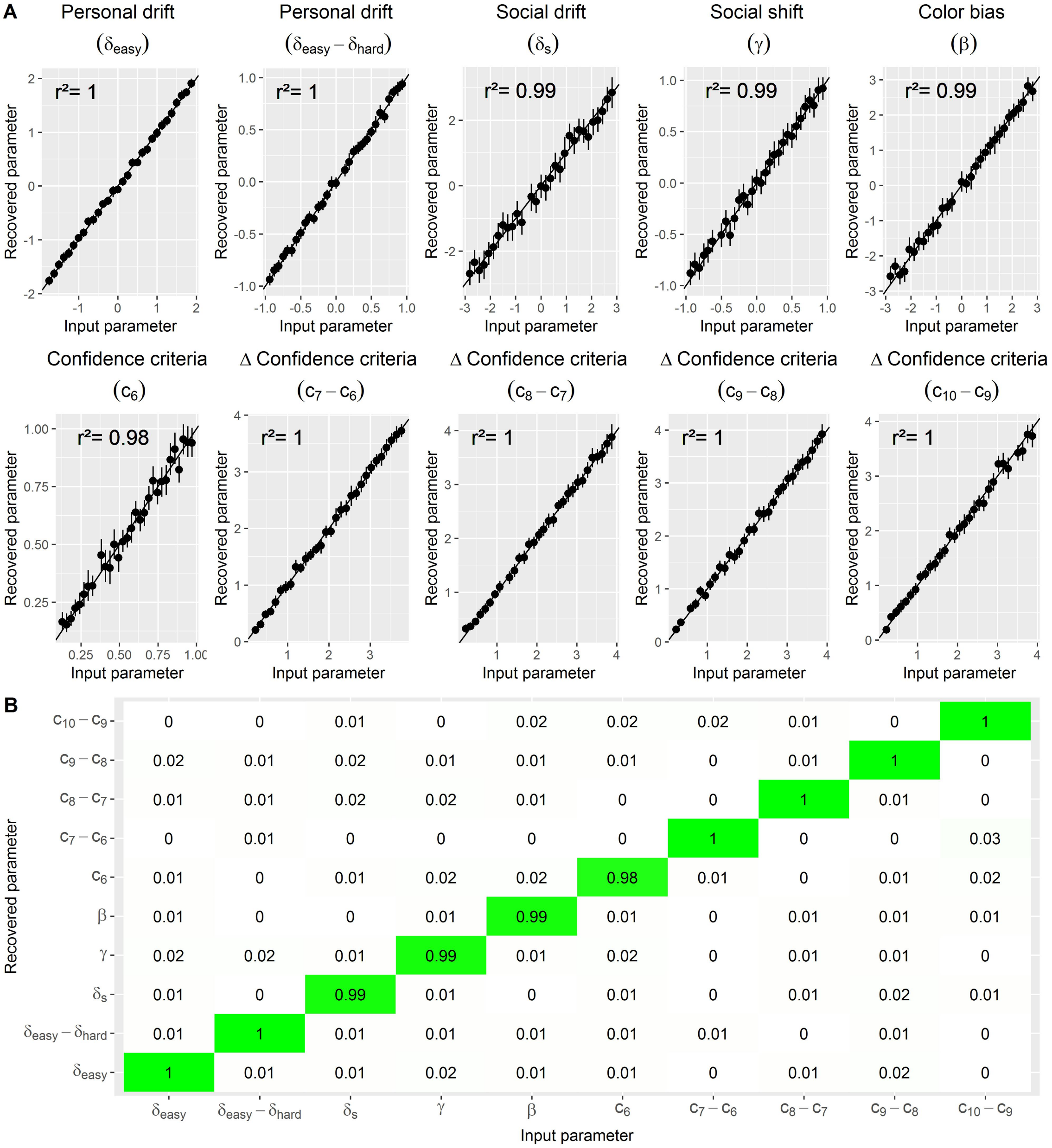

Figure S3. Parameter recovery analysis. (a) Relationship of the input parameters (x-axis) and the recovered parameter estimates. (b) Correlation matrix of input parameters and recovered parameter estimates. There was a strong positive correlation between the generated and the recovered parameter for all parameters, but not across different parameters. The parameters are thus interpretable and capture distinct processes. To conduct the recovery analysis, we repeatedly (30 times) generated data with random input parameters and recovered them with the same hierarchical model used to analyse the empirical data. The input parameters were sampled with a quasirandom number generator (using the sobol sequence), ensuring an even distribution across a large multidimensional parameter space. Using these input parameters, we sampled confidence judgments from computed probabilities that take the stimulus difficulty and social information characteristics (presence, validity, and timing) observed by the participant at a given trial into account. The generated data thus have the same hierarchical structure as the empirical data in Experiment 1, with 99 participants, each conducting 100 trials (excluding filler trials). We report the mean of the posterior distributions and the 95% CI of the higher order group-level estimate. To measure the relationship between input and recovered parameters, we calculated the square of the Pearson correlation coefficient r^2 for each parameter combination.

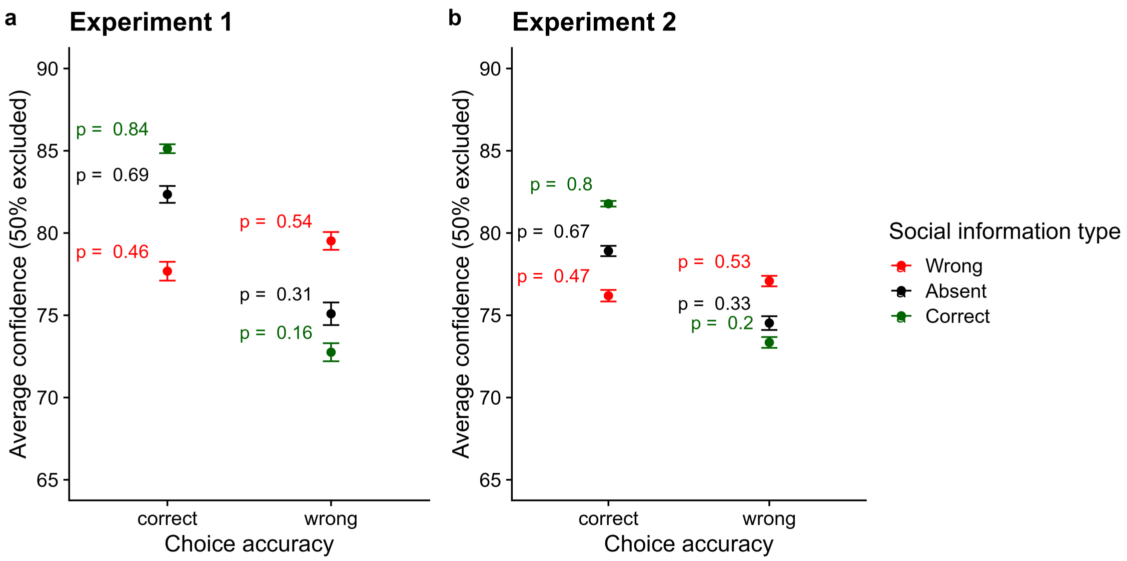

Figure S4. The average reported confidence on a scale from 60% to 100% for different social information conditions (50% confidence was excluded because no choice can be assigned to it). The numbers indicate the proportion of choices within a social information condition. Error bars indicate standard error.

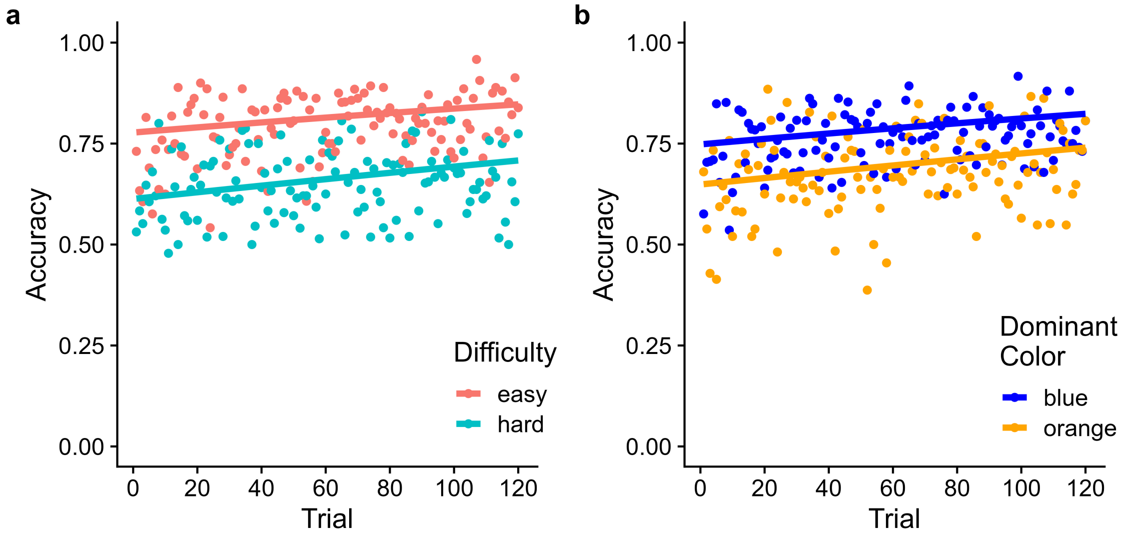

Figure S5. Accuracy over time in Exp. 1. Accuracy over time by (a) difficulty level and (b) dominant color. Dots show the raw means; lines show the regression model predictions by treatment.

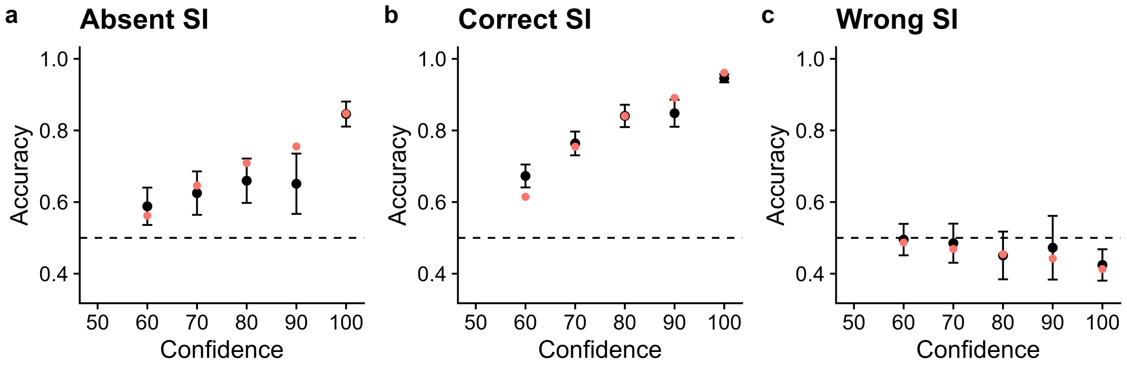

Figure S6. **Confidence–accuracy relationship in Experiment 1**. (a) There was a positive confidence–accuracy relationship in trials without social information (SI). This relationship (b) was stronger in trials with correct social information but (c) disappeared and tended to reverse in trials with wrong social information. Black dots indicate raw means; error bars indicate twice the standard error. Red dots indicate accuracy predicted by the signal detection analysis.

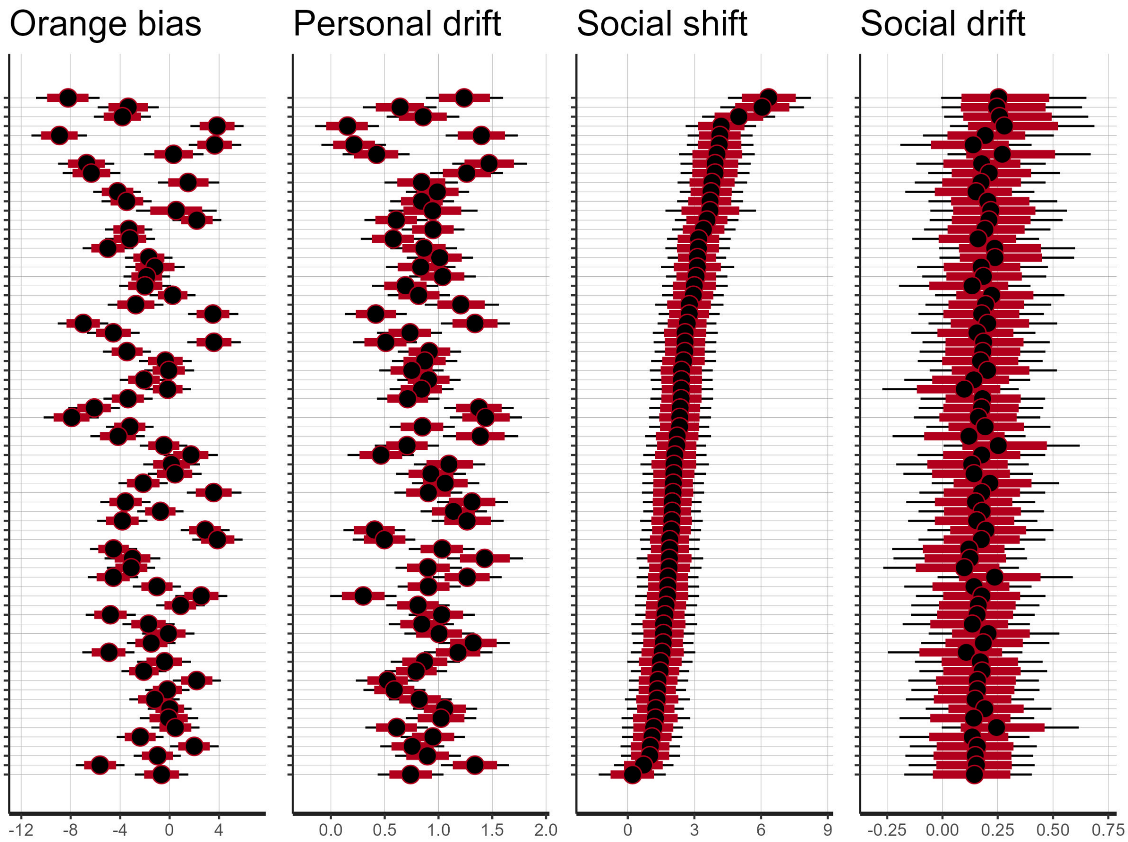

Figure S7. **Posterior estimates of all subjects from Experiment 1**. The black dots indicate the average parameter estimates for each subject for color bias (positive values indicate an orange bias), personal drift, social shift and social drift (see Table 1 for a description of parameters). The red and black error bars indicate the 80% and 95% credibility intervals. The estimates were ordered according to the strength of the social shift.

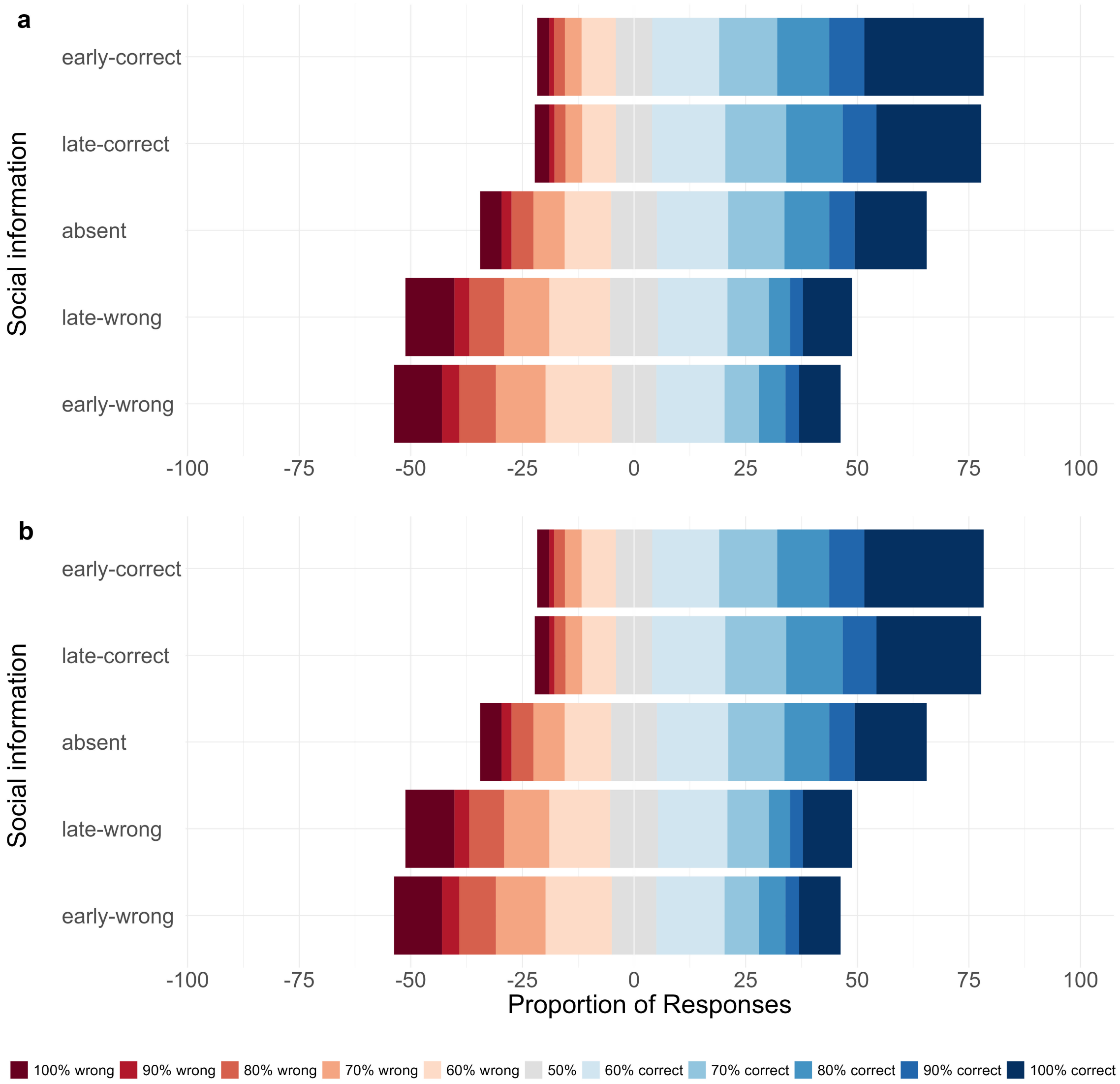

Figure S8. Distribution of confidence judgments as (a) observed in Exp. 1 and (b) predicted by the cognitive model in Exp. 1. Proportions of confidence judgments by treatment. Blue/red color shows proportions of confidence judgments for correct/wrong choices. Gray indicates neutral choices (i.e., 50%). The cognitive model reproduces the empirical patterns well, including the positive/negative influence of correct/wrong social information and the stronger influence of early social information.

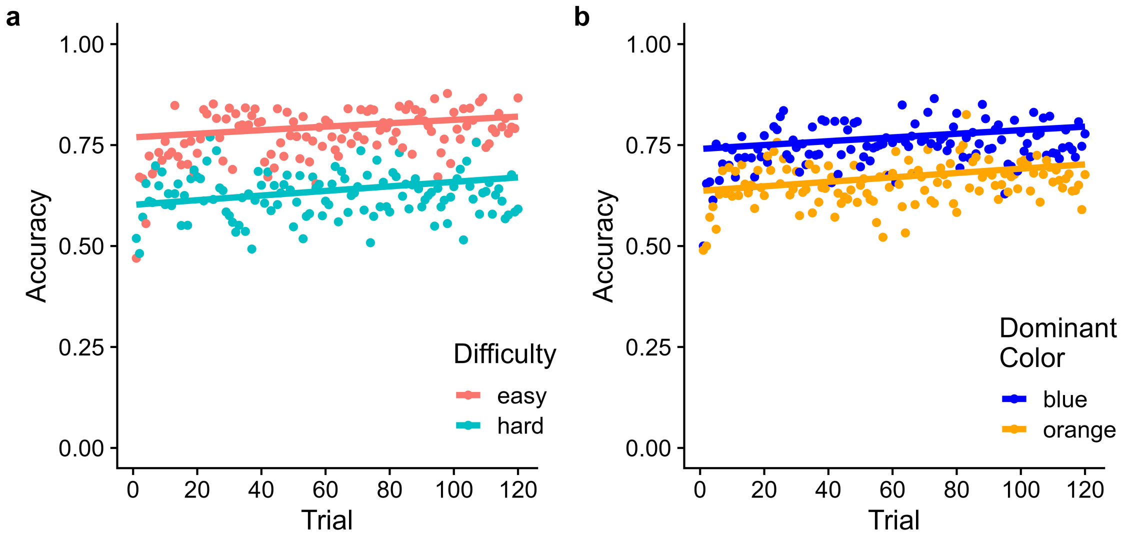

Figure S9. Accuracy over time in Exp. 2. Accuracy over time by (a) difficulty level and (b) dominant color. Dots show the raw means; lines show the model predictions per treatment.

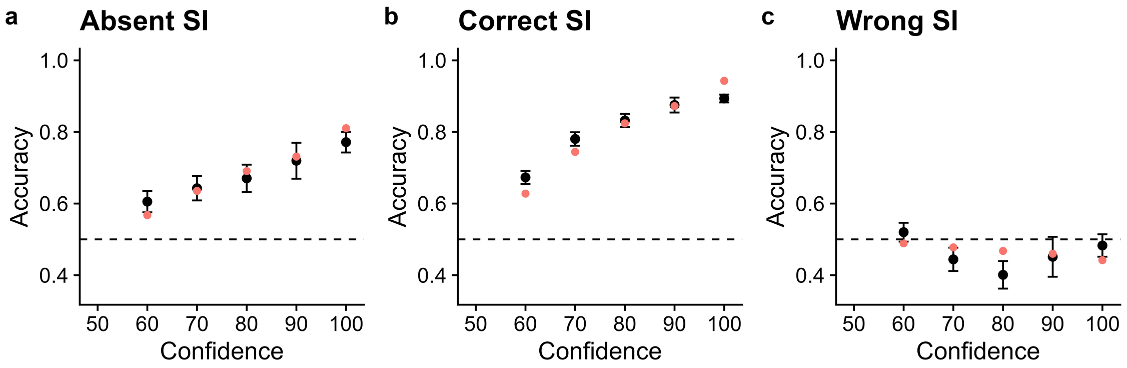

Figure S10. Confidence–accuracy relationship in Experiment 2. (a) There was a positive confidence–accuracy relationship in trials without social information (SI). This relationship (b) was stronger in trials with correct social information but (c) tended to reverse in trials with wrong social information. Black dots indicate raw means; error bars indicate twice the standard error. Red dots indicate accuracy predicted by the signal detection model.

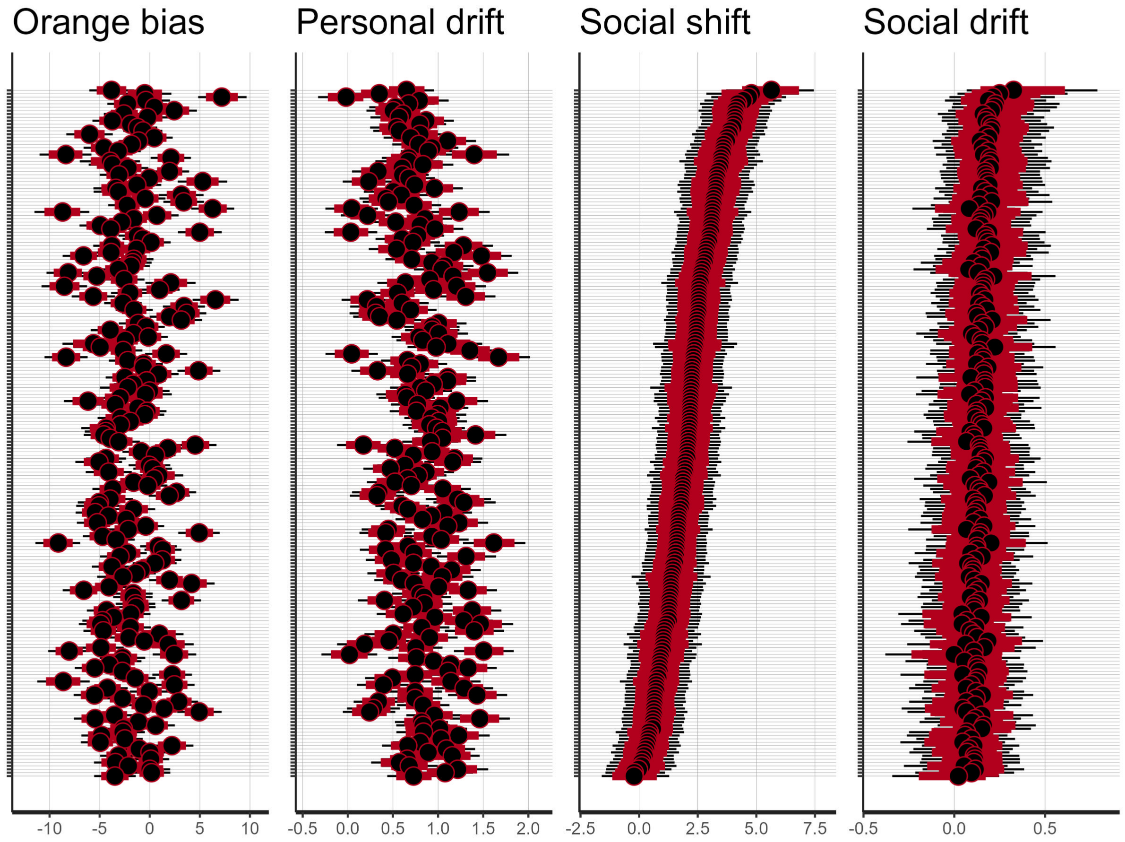

Figure S11. Posterior estimates of all subjects from Experiment 2. The black dots indicate the average parameter estimates for each subject for color bias (positive values indicate an orange bias), personal drift, social shift and social drift (see Table 1 for a description of parameters). The red and black error bars indicate the 80% and 95% credibility intervals. The estimates were ordered according to the strength of the social shift.

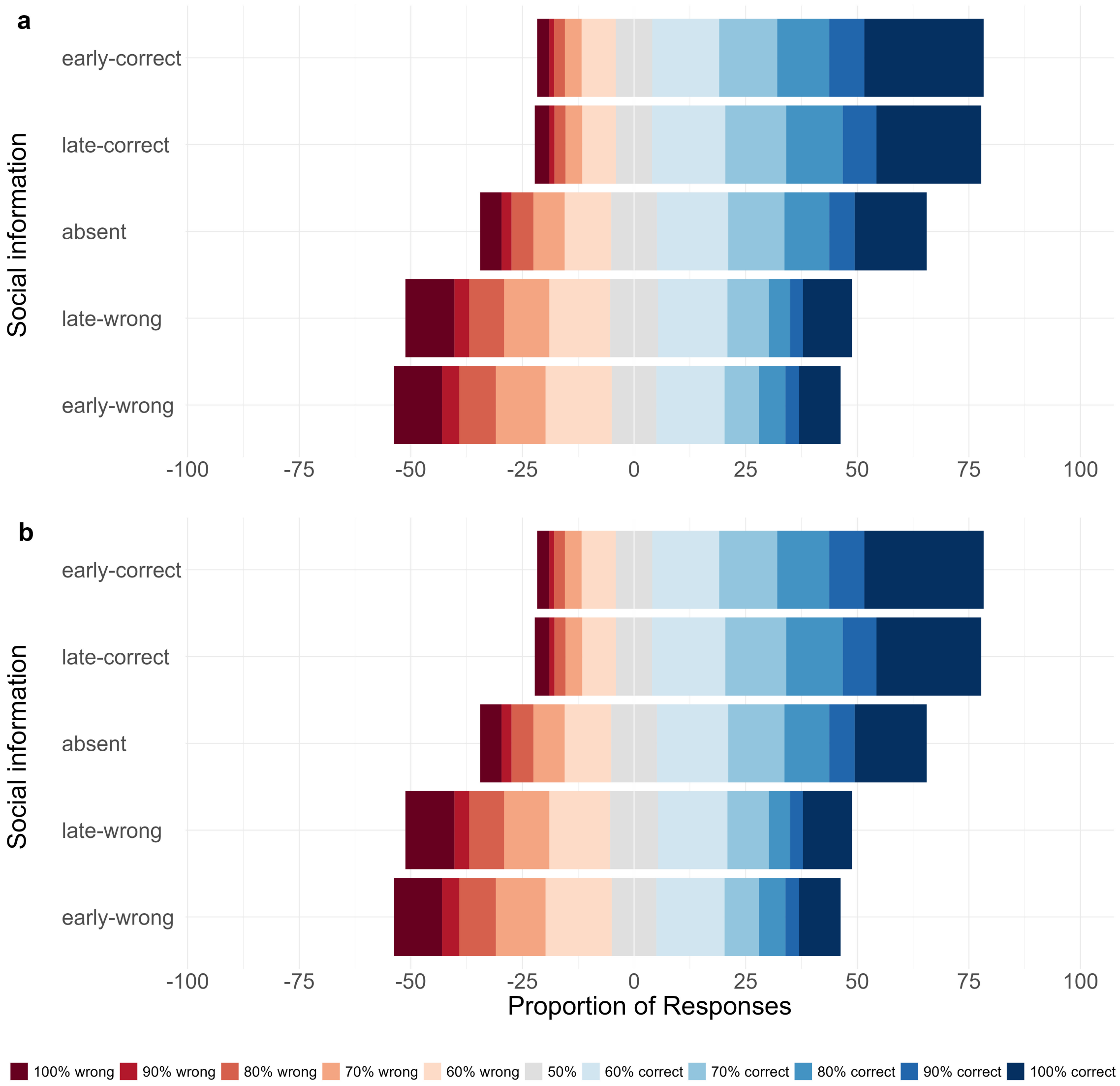

Figure S12. Distribution of confidence judgments as (a) observed in Exp. 2 and (b) predicted by the cognitive model in Exp. 2. Proportions of confidence judgments by treatment. Blue/red color shows proportions of confidence judgments for correct/wrong choices. Gray indicates neutral choices (i.e., 50%). Again, the cognitive model reproduces the empirical patterns well.
